# ATP1A3-Related Disorders: An Ever-Expanding Clinical Spectrum

**DOI:** 10.3389/fneur.2021.637890

**Published:** 2021-04-01

**Authors:** Philippe A. Salles, Ignacio F. Mata, Tobias Brünger, Dennis Lal, Hubert H. Fernandez

**Affiliations:** ^1^Department of Neurology and Center for Neurological Restoration, Neurological Institute, Cleveland Clinic, Cleveland, OH, United States; ^2^Centro de Trastornos del Movimiento, Centro de Trastornos del Movimiento (CETRAM), Santiago, Chile; ^3^Cleveland Clinic Foundation, Genomic Medicine, Lerner Research Institute, Cleveland, OH, United States; ^4^Cologne Center for Genomics, University Cologne, Cologne, Germany

**Keywords:** ATP1A3, sodium-potassium-exchanging ATPase, rapid-onset dystonia parkinsonism, Dyt12, alternating hemiplegia, CAPOS syndrome, ataxia

## Abstract

The Na+/K+ ATPases are Sodium-Potassium exchanging pumps, with a heteromeric α-β-γ protein complex. The α3 isoform is required as a rescue pump, after repeated action potentials, with a distribution predominantly in neurons of the central nervous system. This isoform is encoded by the ATP1A3 gene. Pathogenic variants in this gene have been implicated in several phenotypes in the last decades. Carriers of pathogenic variants in this gene manifest neurological and non-neurological features in many combinations, usually with an acute onset and paroxysmal episodes triggered by fever or other factors. The first three syndromes described were: (1) rapid-onset dystonia parkinsonism; (2) alternating hemiplegia of childhood; and, (3) cerebellar ataxia, pes cavus, optic atrophy, and sensorineural hearing loss (CAPOS syndrome). Since their original description, an expanding number of cases presenting with atypical and overlapping features have been reported. Because of this, ATP1A3-disorders are now beginning to be viewed as a phenotypic continuum representing discrete expressions along a broadly heterogeneous clinical spectrum.

## Introduction

Considered rare, *ATP1A3*-related disorders have been capturing our attention in the last decade by virtue of cumulative cases reporting an expanding range of clinical and genetic variability. In the same manner, next-generation sequencing technologies have arisen fulfilling a major role in the understanding of the genotype-phenotype association of these newfangled syndromes. These have been discussed by the authors of this article in a recent editorial (1).

The Na^+^/K^+^ ATPase is a transmembrane ion-pump located at the cellular plasma membrane. This pump extrudes three Na^+^ and import two K^+^ into the cell for every adenosine triphosphate (ATP) split. Its main role is to regulate electrochemical gradients, and it is involved in the action potential propagation during neuronal depolarization.

The Na^+^/K^+^ ATPase is a heterotrimeric α-β-γ protein complex. Humans express four α isoforms (α1–4), encoded by the ATP1A 1-4 genes, respectively (2). The α3 isoform, encoded by *ATP1A3* located on chromosome 19q, is expressed almost exclusively in neurons (3). This isoform is specifically required as a rescue pump, after repeated action potentials, for rapid restoration of large transient increases in intracellular Na^+^ concentration (4). Conditions associated with α3 deficiency are therefore likely aggravated by supra-threshold neuronal activity. The α3 isoform has been also suggested to support re-uptake of neurotransmitters (3, 5).

In the adult mouse brain Bøttger et al. found high expression of the Na^+^/K^+^ -ATPase1 α3 isoform in the striatum, globus pallidus, subthalamic nucleus, substantia nigra, thalamus, cerebellum, red nucleus, oculomotor nucleus, reticulo-tegmental nucleus of pons, and hippocampus, mainly in co-location with GABAergic neurons (6). In the retina, photoreceptor and all neuronal-type cells express Na^+^/K^+^ -ATPase1 α3 isoform (4). Within the cochlea, it is found in membranes of the spiral ganglion somata and organ of Corti, affecting the innervation pathways of inner hair cell synapses (7).

The main exception for nervous system-specific expression is the cardiac muscle (4).

Familial or most commonly *de novo* heterozygous pathogenic variants of *ATP1A3* are responsible of Na^+^/K^+^ ATPase dysfunction, due to α3 isoform defects. Not surprisingly carriers manifest a range of distinctive neurological syndromes, with some cases presenting atypical manifestations and others overlapping phenotypes.

The causative role of *ATP1A3* variants in the pathogenesis of several neurological disorders with a similar pattern of inheritance has been previously documented in several reviews (8–11). Here we have performed an up-to-date review of this topic, including several novel recently reported phenotypes in *ATP1A3* pathogenic variant carriers.

## Review

### Classical ATP1A3-Related Syndromes

In their original descriptions, the three classic phenotypes related to *ATP1A3* pathogenic variants—rapid-onset dystonia parkinsonism (RDP), alternating hemiplegia of childhood (AHC), and cerebellar ataxia, pes cavus, optic atrophy, and sensorineural hearing loss (CAPOS)—diverge in several clinical features with different pathogenic variants associated with each syndrome. However, in more recent years these pragmatic limits are less clear. In this section, we describe these syndromes in the chronological order of their association with *ATP1A3* pathogenic variants.

#### Rapid-Onset Dystonia-Parkinsonism

In 1993 Dobyns et al. reported a previously undescribed “rapid-onset dystonia-parkinsonism” (RDP) syndrome with an autosomal dominant inheritance pattern in a large family (12). The association of this syndrome with *ATP1A3* pathogenic variants was made by De Carvalho and colleagues in 2004. This was the first disorder that was found to be caused by variants in *ATP1A3* (13). Pathogenic variants presenting as RDP are evenly distributed throughout the *ATP1A3* gene (8). Nowadays p.Thr613Met is known to be the most common pathogenic variant in RDP (14).

RDP is an autosomal dominant disorder with variable penetrance, although some cases may appear sporadic due to *de novo* pathogenic variants, with an onset most commonly in the teens to twenties (15). Approximately half of the pathogenic variants occurred *de novo* (16).

Typically RDP debuts with an abrupt onset, and a limited progression over weeks. Usual manifestations include: bulbar symptoms (generally dysarthria and hypophonia with mild to moderate dysphagia), cranio-cervical dystonia, mild limb dystonia, and parkinsonism (mainly bradykinesia and postural instability) with no pill-rolling tremor, diurnal fluctuation, nor response to L-dopa.

It is habitually triggered by a physical or psychological stressor such as exercising, alcohol binges, minor head injuries, overheating, emotional stress, infections, or childbirth (8).

Before the onset of RDP, several patients report vague symptoms of dystonia associated with bradykinesia, typically lasting for hours to days (15). In the majority, this was mild and confined to the distal arm or leg. Generalized or truncal dystonia was never reported as a preceding symptom. Several cases were rarely followed by abrupt exacerbations, occurring 1–9 years after the initial onset. Seizures may rarely appear several years later (12, 15).

Initially RDP was considered a well-defined stereotypical phenotype associated to certain *ATP1A3* variants, with a nearly non-overlapping set of pathogenic variants associated with AHC or RDP. However, RDP phenotypical variability has been reported among non-related carriers of the same pathogenic variant, and even among individuals of the same family. More recently intermediate RDP-AHC phenotypes with a genotype-phenotype overlapping have also been reported (15, 17).

Psychiatric symptoms are common. Bipolar disorder, dysthymia, and agoraphobia have been reported (18). Across different families with distinct *ATP1A3* pathogenic variants, Brashear et al. found higher prevalence of mood disorders and psychosis in patients with RDP who had motor symptoms, compared to controls and non-motor manifesting carriers (19). Cognitive impairment, especially verbal learning and memory, non-contextual visual memory, processing speed, attention, and executive functioning appear to be part of RDP syndrome (20).

Brashear and colleagues proposed the minimal clinical criteria for RDP (15):

1. Abrupt onset of dystonia with features of parkinsonism over a few minutes to 30 days.
2. A clear rostro caudal (face > arm > leg) gradient of involvement.
3. Prominent bulbar findings.

In addition, other features suggestive of RDP include:

4. Minimal or no tremor at onset.
5. Occasional mild limb dystonia prior to the primary onset of RDP.
6. Common reports of triggers associated with the abrupt onset of symptoms.
7. Rare “second onsets” or abrupt worsening of symptoms later in life.
8. Stabilization of symptoms within a month.
9. Minimal improvement overall but with limited improvement in gait (seen in a few patients).

Investigations of a large cohort allowed Haq et al. to show that not all of the considered classical features of RDP are really characteristic, and that even characteristic features may be absent (16). Remarkably, rapid onset and bulbar predominance was not universally present in pathogenic variant carriers. Non-rapid onset (over more than 30 days) was the clinical onset in about 20% of cases. Arms were the first body part affected (41%), followed by legs (21%), and face (2%). At longer follow-up, arms and voice were most severely affected. A strict rostro-caudal gradient of dystonia severity was present in only 7% of carriers, while parkinsonism was strongly correlated with dystonia (16).

Atypical signs have been reported in RDP, including prominent lower limb dystonia, late age of onset (>50 years of age) (18); dystonia without signs of parkinsonism, typical writer's cramp (12); pyramidal signs (p.Glu277Lys) (21); Myoclonus, ataxia, chorea (22, 23); and hyporeflexia (23).

For a comparison of the main clinical features of RDP with other classical syndromes see Table 1.

**Table 1:** Clinical characteristics of ATP1A3 classical syndromes.

	**AHC**	**CAPOS**	**RDP**
Age of onset	Before 18 months of age	Infancy-childhood (Frequently between 1 and 5 years)	After 18 months (often second to third decade)
Triggers	Fever, stress, excitement, extreme heat or cold, water exposure, physical exertion, lighting changes, and foods	Fever	Fever, running, alcohol binges, minor head injuries, overheating, emotional stress, infections, sleep deprivation, or childbirth
Onset	Acute onset	Acute-subacute ataxia	Abrupt onset over a few minutes to 30 days.
Typical manifestation	- Hemiplegia or quadriplegia - Tonic or dystonic crises - Oculomotor abnormalities - Cognitive impairment - autonomic phenomena	- Early onset cerebellar ataxia with a relapsing course - Areflexia - Pes-cavus - Optic atrophy - Sensorineural hearing loss.	- Bulbar and limb dystonia - Parkinsonism. - Rostro caudal gradient
Precede symptoms	Paroxysmal ocular manifestations, seizures, developmental delay	Fever-induced transient encephalopathy	Vague symptoms of dystonia in distal limbs
Course	Polyphasic (Relapsing-remitting)	Relapsing course of ataxia-encephalopathy (one to three episodes) with slow progression of other features	Rarely “secondary” exacerbations (2–3 episodes) occurring 1–9 years after the initial onset
Atypical manifestation	Benign familial nocturnal AHC; Mild AHC; Dystonia-predominant AHC, Familial dominant pedigree; Late-onset AHC; AHC without quadriparesis	Urinary urgency; Cardiac arrhythmia, left ventricular enlargement; Scoliosis; Cognitive dysfunction; Autistic traits; Bradykinesia; Myoclonus, Chorea, Tremor, Oral dyskinesias; Dystonia	Prominent lower limb dystonia; Late onset (>50 years of age); Gradual onset; Pure dystonia; Writer's cramp; Pyramidal signs; Myoclonus; Ataxia, Chorea; Hyporeflexia.

*AHC, alternating hemiplegia of childhood; CAPOS, cerebellar ataxia, areflexia, pes-cavus, optic atrophy, sensorineural hearing loss; RDP, rapid onset dystonia parkinsonism*.

Brashear and colleagues reported in 2012 a novel phenotype in two unrelated children with onsets at age 9 months and 4 years, respectively. The former carrying the *ATP1A3* p.Arg756His and the later the p.Asp923Asn variant. Case 1's initial symptoms were three episodes of intermittent flaccidity preceded by illness with and without fever. Case 2 had a baseline history of hypotonia with superimposed spells of flaccidity and bulbar symptoms before sudden onset of dystonia of the limbs. Subsequently both patients developed bulbar symptoms including severe dysarthria and dysphagia, which is more characteristic of RDP (24).

Gradual onset of dystonia and parkinsonism has been reported (25). An insidious onset of asymmetrical parkinsonism evolving over a year, remaining stable for ~3.5 years before an acute episode of bulbar signs with oromandibular dystonia and more severe parkinsonism in a 38 year-old p.Ile274Thr carrier was reported (26).

Decreased CSF levels of homovanillic acid have been inconsistently reported. Dopamine pre-synaptic SPECT imaging has been normal in all cases, thus supporting anatomo-pathological data of intact nigro-striatal neurons in RDP (27).

#### Alternating Hemiplegia of Childhood

Alternating hemiplegia of childhood (AHC) was first defined by Verret and Steele as a distinct syndrome in 1971, in a report that described eight patients with episodes of intermittent hemiplegia on alternating sides of the body, developmental delay, dystonia, and choreoathetosis beginning in infancy (28).

Onset usually occurs before the age of 6 months. In a cohort of 157, hemiplegic attacks were always present usually involving ipsilateral limbs, with face generally spared; 86.5% reported episodes of bilateral weakness without pyramidal signs; 88% with dystonic attacks involving one or more limbs, occurring alone or mixed with hemiplegic episodes, rarely involving the tongue; 49% with events of autonomic dysfunction; 53% with epilepsy; 72% developed chorea and/or dystonia; and 92% had developmental delay. Abnormal ocular movements, often monocular nystagmus, and hypotonia were common and tend to regress into adulthood (29).

Common triggers include stress, excitement, extreme heat or cold, water exposure, physical exertion, lighting changes, and foods (8).

AHC diagnostic criteria were proposed by Neville in 2007. Typical cases satisfied criteria 1, 2, 3, and 7 (30):

1. Onset of symptoms before 18 months of age.
2. Repeated attacks of hemiplegia involving either side of the body, at least in some episodes.
3. Episodes of bilateral hemiplegia or quadriplegia as generalization of a hemiplegic episode or bilateral from the beginning.
4. Other paroxysmal disturbances including tonic or dystonic crises, oculomotor abnormalities (e.g., strabismus or nystagmus), and autonomic phenomena occurring during hemiplegic episodes or in isolation.
5. Immediate disappearance of symptoms upon sleeping, which later may resume, usually 10–20 min after waking.
6. Evidence of developmental delay and neurological abnormalities including choreoathetosis, dystonia, or ataxia.
7. Not attributable to another disorder.

The incidence of AHC is about one in one million individuals (30).

In 2012 Heinzen and colleagues, and Rosewich and colleagues reported heterozygous variants in *ATP1A3* associated with AHC (31, 32).

*De novo ATP1A3* pathogenic variants explain the majority of patients with AHC (32).

Variants presenting with AHC are clustered within certain regions of that gene. Typically, AHC occurs in carriers of variants in an amino acid position before 400 or above 800 (8).

The most frequent *ATP1A3*pathogenic variants causing AHC are p.Asp801Asn, p.Glu815Lys, and p.Gly947Args. The former variant accounting for up to 43% of all *ATP1A3*-related AHC cases (8).

In particular, p.Glu815Lys (16–35% of cases) is associated with a severe intellectual and motor disability, high prevalence of epilepsy with early onset of seizures and poor prognosis, whereas p.Asp801Asn (30–43% of cases) results in a moderate/mild form of the disease, and p.Gly947Arg (8–15% of cases) has a favorable prognosis (33).

Unlike *ATP1A3* variants that cause RDP, AHC-causing variants in this gene cause consistent reductions in ATPase activity without affecting the level of protein expression (31).

Cerebellar vermian atrophy has been observed (34). A brain magnetic resonance spectroscopy showed an increase time in choline and in lipids at the pons region, with normal NAA levels at the age of 40 and 44 years, similar to those findings observed in chronic inflammatory or hypo-myelinating CNS disorders (34).

Several atypical AHC cases have been reported, including benign familial nocturnal AHC, mild AHC, dystonia-predominant AHC, familial autosomal dominant pedigree, late-onset AHC, and AHC without quadriparesis (35).

A patient with p.Ser137Phe and AHC developed in her twenties episodes of loss of consciousness related to recurrent periods of asystole up to 5 s long, which required a peacemaker (36).

Gurrieri et al. found that 22 of 26 AHC patients with confirmed *ATP1A3* pathogenic variants shared a similar physical phenotype consisting of generalized hypotonia, long face, thin and well-defined eyebrows, strabismus, widely spaced eyes, long palpebral fissures, downturned mouth, and slender habitus. Authors considered this phenotype sufficiently typical to delineate a recognizable phenotype (37).

#### CAPOS Syndrome

In 1996 Nicolaides, Appleton and Fryer described three members of a family affected by a likely dominantly inherited syndrome characterized by early onset cerebellar ataxia with a relapsing course, areflexia, pes cavus, optic atrophy, and sensorineural hearing loss. They encompassed this association under the acronym of “CAPOS” (38). It was not until 2014 that Demos and colleagues found a heterozygous missense *ATP1A3* variant, p.Glu818Lys, in individuals from two independent families manifesting CAPOS syndrome (39).

Heimer and colleagues described in 2015 a phenotype highly resembling the patients described by Demos et al. except for the lack of pes cavus. They identified the same heterozygous p.Glu818Lys variant in the *ATP1A3* gene. They named this syndrome CAOS (Episodic Cerebellar Ataxia, Areflexia, Optic Atrophy, and Sensorineural Hearing Loss) (40) and remarked that pes cavus was found only in 3 of 10 patients described by Demos et al. Since the prevalence of pes cavus in the general population is ~10% and because pes cavus was also absent in 7 patients in their cohort, it might be an incidental finding rather than a key feature of the disorder (40).

So far, more than 50 CAPOS or CAOS patients with *ATP1A3* p.Glu818Lys have been reported (41).

Roenn and colleagues characterized the functional defects of the CAPOS *ATP1A3* p.Glu818Lys using a combination of biochemical and electrophysiological measurements, which allowed demonstration of a reduced Na^+^ affinity of the transport sites of the CAPOS mutant in internally as well as externally facing conformations. Consequently, the CAPOS mutant pump may fail to clear the neuron fast enough of the accumulated Na^+^ in relation to action potentials, and this defect might be part of the pathophysiological mechanism (41).

CAPOS/CAOS frequently starts between 1 and 5 years of age, with a fever-induced, acute-onset cerebellar ataxia, accompanied by encephalopathic features, disturbed eye movements, hypotonia, areflexia, and mild weakness. Other less common episodic symptoms include paresis (hemi/para/tetra paresis), transient hearing and visual loss. Most patients have a complete recovery, although persistent ataxia is not rare. A relapsing course is characteristic (10, 42). Patients classically manifest two to three episodes before transitioning to a slowly progressive evolution (42).

Sensorineural hearing loss with a sudden-onset and progressive nature is a distinctive disabling feature of CAPOS syndrome (43). Han and colleagues reported 3 sporadic cases of auditory neuropathy spectrum disorder (ANSD) with an onset after language acquisition. Interestingly two were *ATP1A3* p.Glu818Lys carriers. The first proband did not manifest any features of CAPOS, while the second proband was compatible with a CAPOS syndrome (44).

Progressive loss of vision, with poor color discrimination and diminished brightness sensitivity and bilateral optic disc atrophy indicative of optic neuropathy are expected findings (39), and very rarely may be absent (42).

Nystagmus and strabismus are also frequent characteristics in long term follow-up (42).

Areflexia is always present, with normal nerve conduction velocities (NCVs) reported (38, 45). Nerve biopsy may reveal findings consistent with axonal neuropathy (39).

Less common manifestations in CAPOS/CAOS syndrome include urinary urgency, cardiac arrhythmia, left ventricular enlargement, scoliosis, cognitive dysfunction, autistic traits (e.g., repetitive behaviors and social difficulties), bradykinesia, myoclonus, chorea, tremor, oral dyskinesias, and dystonia (39, 42, 46).

Dystonia has been reported as (1) cervical dystonia with dystonic tremor responsive to onabotulinumtoxinA (39); (2) transient upper limb dystonia with acute onset at 20 months of age, evolving years later with persistent limb dystonia (45); (3) multi-focal upper limb dystonia (42); and (4) slight focal hand dystonia-myoclonus (42).

The coexistence of CAPOS syndrome and hemiplegic migraine with *ATP1A3* p.Glu818Lys was made by Potic and colleagues (47).

Stagnaro and colleagues reported two cases, with the same *de novo* p.Arg756Cys pathogenic variant, of paroxysmal “CAPOS-like” symptoms, based on areflexia and ataxia, accompanied by dystonia and hypotonia, related to febrile episodes. However, they did not report optic atrophy or sensorio-neural hearing loss (48).

A comparison of the clinical features of the 3 classic *ATP1A3*-related syndromes is presented in Table 1.

### Non-classical Phenotypes

Recently, an increase in the number of “non-classical” phenotypes have been reported.

#### Relapsing Encephalopathy With Cerebellar Ataxia

Dard and colleagues in 2015 reported a novel phenotype in a 34-year-old woman, caused by heterozygous *ATP1A3* p.Arg756Cys variant, consisting of a relapsing encephalopathy during febrile illnesses, accompanied by a prominent cerebellar syndrome, generalized dystonia, pyramidal signs, and anger outbursts. The acronym RECA (relapsing encephalopathy with cerebellar ataxia) was proposed (14).

Hully and colleagues in 2017 reported a family with two affected siblings in whom mosaic heterozygous *ATP1A3* p.Arg756Cys variant was identified.

The first infant started at the age of 9 months, after a febrile episode, with severe psychomotor regression with subsequent developmental delay. Later she developed abnormal ocular movements with severe cerebellar ataxia, choreic, and dystonic movements. Her sister, had a normal early development until the age of 22 months when she regressed during a febrile episode with acute ataxia, pyramidal signs, and hypotonia. Subsequently, she developed severe encephalopathy with cerebellar ataxia combined with nystagmus, and dystonic movements with bucco-facial involvement. She experienced two additional relapses during febrile infections at the ages of 4 and 6. Her MRI showed mild cerebellar atrophy (49).

Later in 2019 Sabouraud and colleagues described eight new RECA pediatric cases, associated with *ATP1A3* p.Arg756Cys (50).

#### Fever-Induced Paroxysmal Weakness and Encephalopathy

Yano and colleagues in 2017 grouped patients with *ATP1A3* p.Arg756His or *ATP1A3* p.Arg756Leu pathogenic variants, with clinical onset before the age of 3 years. Fever-Induced Paroxysmal Weakness and Encephalopathy (FIPWE) was the main phenomenon, accompanied by different combinations of oculomotor abnormalities, dysphagia, generalized hypotonia, dystonia, ataxia, or apnea (51).

#### Early Life Epilepsy

Paciorkowski et al. in 2015 reported two cases presenting with epilepsy. One was a child with catastrophic early life epilepsy (EE), who seized 4 h after birth. Her epilepsy continued to be intractable with recurrent episodes of status epilepticus. MRI showed progressive brain atrophy and postnatal microcephaly. She died at 16 months. The second patient had epilepsy after 6 weeks of age. His seizures were characterized by episodic, prolonged apnea, and gaze deviation. He developed postnatal microcephaly and severe developmental disability. *ATP1A3* p.Gly358Val and p.Ile363Asn were identified in these children (52).

Marzin et al. reported three children with *de novo* p.Asp742Tyr, p.Cys346Arg, and p.Asp609Tyr variants in *ATP1A3*, respectively, manifesting features close to those cases reported by Paciorkowski et al. with early-onset encephalopathy, seizures and non-epileptic attacks of movement disorders, mainly observed during infancy. No obvious plegic attacks neither mycrocephaly, were observed in these cases (53).

Holze and colleagues reported two girls presenting with unexplained severe apneic episodes around the first year of life. One patient was *ATP1A3* p.Gly89fs carrier and the other had the p.Gly706Arg variant. The authors hypothesized that the apneic episodes were symptoms of *ATP1A3*-related early onset epilepsy (54).

Hully and colleagues in 2017 reported two affected siblings in whom mosaic heterozygous *ATP1A3* p.Gly706Arg variant was identified. The siblings had neurodevelopmental delay, and seizures of varying semiology starting at 4.5–2 months of age, evolving to severe encephalopathy with autistic features, epilepsy, strabismus, nystagmus, pyramidal signs, dystonic, and ataxic gait. Brain MRI performed showed bilateral hippocampus sclerosis and cerebellar atrophy (49).

An infant carrier of trinucleotide deletion *ATP1A3* p.Asp756del manifesting at the age of 3 months with drug-resistant epileptic encephalopathy responsive to ketogenic diet, plus non-epileptic paroxysmal episodes (hypotonia, hemiplegia, apnea, monocular nystagmus) and developmental delay was reported (55).

Tran and colleagues reported a p.Val589Phe carrier whose initial presentation was an epileptic encephalopathy starting at the age of 4 months, with subsequent AHC and then RDP symptomatology (56).

#### Rapid Onset Cerebellar Ataxia

Adult rapid onset cerebellar ataxia (ROA) phenotype was reported by Kathleen and colleagues in 2016. A male with normal development except for mild amblyopia, learning disability, and dyslexia, presented at age 19 with episodes of vertigo lasting for days. At age 21, he developed ataxia, progressing over 6 months requiring the use of a wheelchair. Progressive cerebellar degeneration was evident on MRI. Follow-up evaluation at age 26 revealed partial overlap with RDP syndrome. A novel *ATP1A3* p.Gly316Ser variant was identified (57).

Gusmao and colleagues reported a 28-year-old *ATP1A3* p.Gly316Ser carrier with a history of mild learning disability and migraines who developed falls at 21 years. There were no clear triggers. This progressed to prominent gait and appendicular ataxia with dysarthria, action tremor, and myoclonus. Oculomotor abnormalities included intrusions and dysmetric saccades. Within a few years, he became wheelchair-dependent. Neuroimaging demonstrated vermian cerebellar atrophy (22).

Schirinzi et al. in 2018 reported three cases of childhood ROA triggered by fever, starting before the age of 20 months. One case had dystonia, while another had self-limited episodes of hypotonus, convergent strabismus, and febrile convulsions. The authors described two different pathogenic variants in *ATP1A3* p.Arg756Cys and p.Glu818Lys, the later commonly associated to CAPOS/CAOS syndrome. These findings reinforced that ataxia may represent a peculiar, sometimes prominent or isolated, feature of *ATP1A3*-related phenotype (58).

#### Slowly Progressive Cerebellar Ataxia

Recently, Sasaki et al. reported two cases presenting with gradually progressive cerebellar ataxia, and mild intellectual disabilities. One of them, beginning with ataxia at the age of 1 year. At the age of 15, his neurological examination revealed intellectual disability, ataxia, and ocular motor apraxia. Brain MRI revealed cerebellar cortical atrophy mainly in the vermis. The other reported case presented early with developmental delay, manifesting later progressive gait unsteadiness and cerebellar atrophy on MRI, since the age of 7 years. None of these patients presented with paroxysmal or episodic symptoms. One patient had the *ATP1A3* p.Met154Val variant, while the other carried the p.Asp350Lys variant (59).

#### Childhood-Onset Schizophrenia/Autistic Spectrum Disorder

Smedemark-Margulies et al. in 2016, reported a case of Childhood-Onset Schizophrenia (COS) with a novel heterozygous pathogenic variant (p.Val129Met) in the *ATP1A3* gene (60). Subsequently in 2017 Chaumette and colleagues identified three *de novo* pathogenic variants in the *ATP1A3* gene (p. Asp801Asn; p.Glu815Lys; p. Ala813Val) in three unrelated individuals with COS, two of them also had AHC. One also had characteristics of autistic spectrum disorder (ASD) (61), which is frequently seen in COS (62).

#### Paroxysmal Dyskinesias

Lastly in 2019 Zúñiga-Ramírez et al. reported a pair of monozygotic twins with interictal mild generalized dystonia and paroxysmal attacks since infancy, resembling paroxysmal non-kinesigenic dyskinesias (PNKD). This attacks were triggered by weather changes, mood swings, caffeine intake, exercise, fever, and infections. Patients also presented speech arrest, and intellectual disability. After excluding known genetic causes of PNKD, whole exome sequencing showed a novel heterozygous *ATP1A3* p.Leu815Arg (63).

Interestingly Roubergue et al. in 2012 reported a family with 3 adult patients with paroxysmal exercise-induced dystonia (PED) but without plegic attacks presenting after childhood. Gene sequencing revealed the heterozygous *ATP1A3* p.Asp923Asn (64).

An *ATP1A3* p.Glu277Lys female carrier was diagnosed with mild intellectual disability, manifesting RDP symptoms when she was 9-year-old. At age of 10 she developed left lower limb paroxysmal dystonia induced by continuous exercise and mentally stressful situations. The frequency of attacks was once every 2 months, lasting 30 min (65).

#### Cerebral Palsy/Spastic Paraparesis

Calame et al. described four non-relatives *ATP1A3* p.Pro775Leu carriers, presenting spastic diplegia, developmental delay, epilepsy, and episodic neurological deterioration. One patient developed also static encephalopathy, microcephaly and dystonia, and one case also had sickle cell disease (66).

#### Dystonia, Dysmorphism, Encephalopathy, MRI Abnormalities, and no Hemiplegia (D-Demø)

Prange et al. reported a distinct phenotype in 4 carriers of *de novo ATP1A3* variants, manifesting with dystonia, dysmorphism of the face, encephalopathy with developmental delay, brain MRI abnormalities always including cerebellar hypoplasia, no hemiplegia (Ø) (D-DEMØ). In these cases, dystonia was triggered by hyperexcitation and/or physiological or psychological stressors, three of these 4 patients presented with seizures. Two presented episodes of quadriplegia, symptoms of dysautonomia, and kyphoscoliosis/scoliosis.

Dysmorphic features included a high forehead with bitemporal narrowing, broad nasal bridge and tip, narrow palpebral fissures, anteriorly facing nostrils, thickened or hypoplastic alae nasi, long philtrum, micrognathia, thin upper lip, prominent lower lip, and incompletely formed antitragus and lower part of the antihelix in the ear pinnae.

In these cases whole-exome sequencing revealed different *ATP1A3 de novo* heterozygous variants: (1) p.Thr360Arg; (2) p.Gln140His; (3) p.Gly325Asp, and 3) p.Glu324Gly (67).

#### Congenital Hydrocephalus

In 2019 Allocco et al. reported one patient with congenital hydrocephalus with aqueductal stenosis, craniosynostosis, open lip schizencephaly, type 1 Chiari malformation, dysgenesis of the corpus collosum, and learning disability. Routine genetic testing (FISH, microarray) was negative. Performing exome sequencing analysis, compound heterozygous *ATP1A3* variants (p.Arg19Cys and p.Arg463Cys) were noted in the exon 2 and 11, respectively, each of which was inherited from one of the patient's unaffected parent (68). Distinctively, these variants are different from the majority of the variants identified in AHC and RDP clustered in exons 8, 14, 17, and 18 (32). Both variants were predicted to be deleterious with a disruptive effect on protein stability. Authors hypothesized that this pathogenic variants can impair CSF homeostasis and thus drive the development of hydrocephalus (68). Two observations supported this hypothesis: (1) Na+/K+-ATPase is known to regulate CSF secretion in the choroid by maintaining an osmotic gradient of Na+; (2) Immunohistochemical studies demonstrate robust ATP1A3 expression in neural stem cells, suggesting a role in regulating neural development (68). Moreover, knockdown of ATP1A3 causes ventriculomegaly in zebrafish (69).

### Overlapping Phenotypes

**Figure 1:**
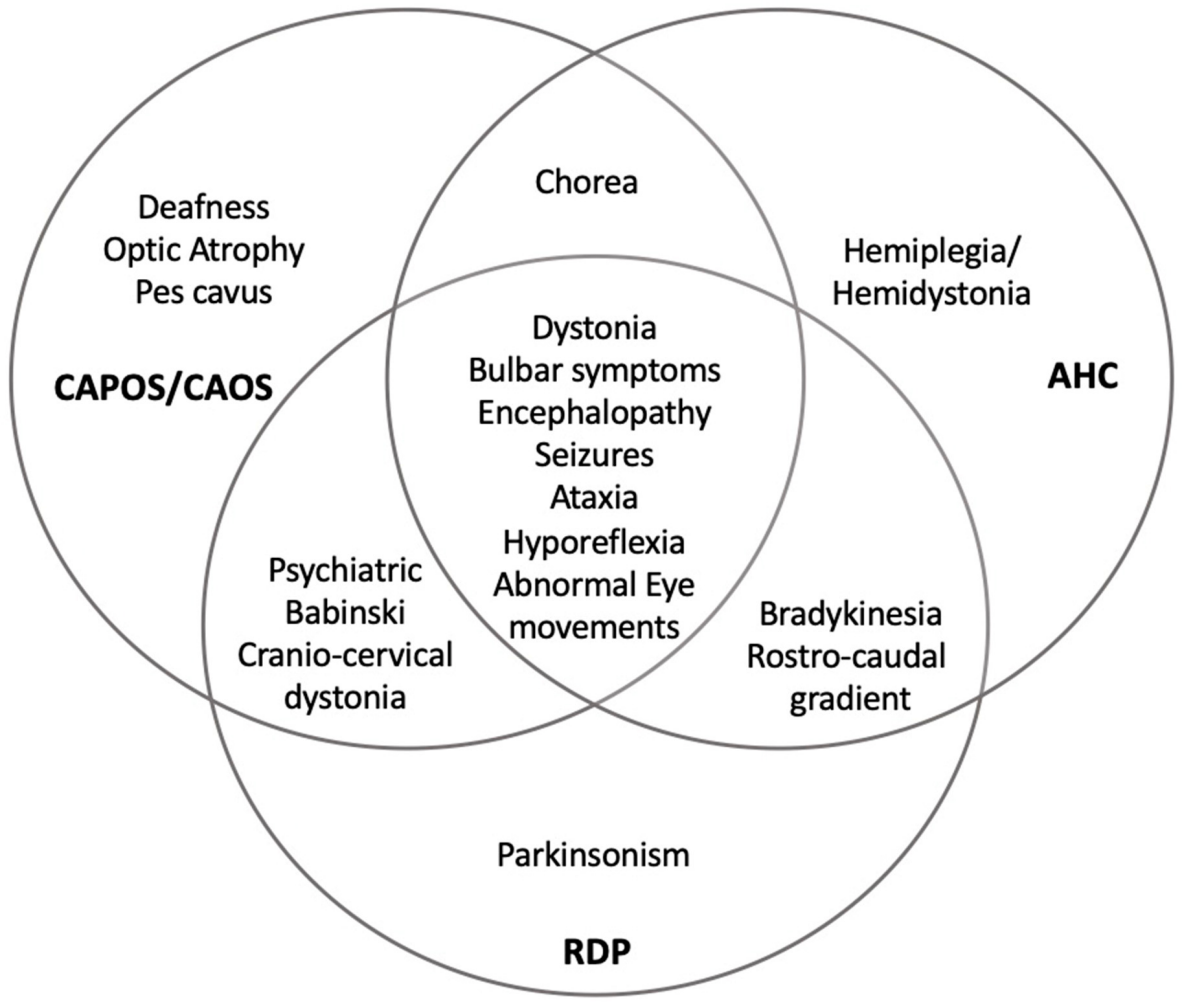
Overlapping of ATP1A3-related disorders. RDP, rapid-onset dystonia-parkinsonism; AHC, alternating hemiplegia of childhood; CAPOS, cerebellar ataxia, areflexia, pes cavus, optic atrophy, and sensorineural hearing loss; CAOS, cerebellar ataxia, areflexia, optic atrophy, and sensorineural hearing loss. With permission from Salles and Fernandez, reference (1).

The three neurological phenotypes RDP, AHC, and CAPOS can present with an acute onset of neurological symptoms triggered by various stimuli. However, their predominant neurological manifestations vary greatly, with early onset hemiplegic/dystonic episodes and developmental delay in AHC, ataxic encephalopathy and impairment of vision and hearing in CAPOS syndrome, and late onset of dystonia/parkinsonism in RDP (3). In addition, intermediate forms and overlapping phenotypes associated with *ATP1A3* are nowadays well-recognized. In Figure 1, we depicted the overlap of manifestations reported for the three classic *ATP1A3*-related syndromes.

Phenotypic overlap of AHC-CAPOS syndrome has been reported in *ATP1A3* p.Glu818Lys carriers. A previously healthy child presented at 20 months with transient afebrile episodes accompanied by abnormal ocular movement, anarthria, generalized hypotonia, paresis-dystonia predominantly of the right arm, and ataxia, with a slow improvement between episodes. At age 6 years after a fever she developed marked and persisting visual impairment. At the age of 12 year old her examination revealed marked dysarthria, bradykinesia, ataxia of gait, muscular hypotonia, mild limbs dystonia, areflexia, optic atrophy, and cochlear hearing impairment (45).

On the other hand, intermediate RDP-AHC presentations have been reported in *ATP1A3* p. p.Asp801Asn, p.Gly867Asp, p.Asp923Asn, p.Glu951Lys, p.Asp583Tyr, and p.Arg756Cys carriers (17, 70–72).

Intermediate CAPOS-RDP cases have been recently described. Chouksey and Pandey reported a case of a 12 year-old girl, who carried a heterozygous *ATP1A3* p.Glu831Lys variant. The symptoms began acutely, with transient lethargy, poor responsiveness, speech problems, and limb posturing after a febrile illness at the age of 18 months. She had a slow recovery after episodes with two relapsing separated by several years. When she was 18 year-old a CAPOS syndrome plus orolingual, cervical, and limb dystonia was established by the authors (73).

Li and colleagues reported a 31 year old man who carried a p.Pro788Leu *ATP1A3* variant. He presented with febrile convulsions yearly since he was 2 year old until the age of 5 years, concomitant with slowly progressive dystonia of the lower limbs. At the age of 26, he fulfilled the criteria for CAPOS syndrome, with atypical features such as a Babinski sign (74).

## Discussion

AHC, RDP, and CAPOS syndrome are considered the prototypical *ATP1A3*-related disorders. Each of these syndromes have particular diagnostic criteria and core features. The genotype-phenotype correlation is variable, with some variants presenting different phenotypes, and particular variants highly correlated with specific syndromes.

Several cases reported in the literature were characterized by overlapping phenotypes with features from the different “classical” phenotypes.

Since the discovery of RDP, AHC, CAPOS syndromes as *ATP1A3* allelic disorders, and with the availability of next-generation sequencing technologies for the genetic diagnosis, an increased number of cases with atypical features or different “non-classical” syndromes have been reported. These novel presentations still present significant clinical and genetic overlapping with the previous reported classical syndromes (for example, p.Glu818Lys in ROA and CAPOS syndrome; p.Glu815Lys in COS/ASD and AHC; or p.Asp801Asn in COS/ASD and AHC/RDP). Some cases still do not fit the reported phenotypes. On the other hand, particular phenotypes have been called with different names by different authors. In the literature RECA and FIPWE have been described as different syndromes, however since these conditions are associated to changes in the same residue (p.Arg756) with largely overlapping phenotypes and just small differences (i.e., apnea and bulbar compromise have been described more frequently in FIPWE than RECA), it appears reasonable to consider these two conditions as part of the same phenotype related to changes in p.Arg756, as other authors have suggested before (50).

It is important to emphasize that some of the novel described phenotypes are based on individual cases so far (i.e., congenital hydrocephalus related to bi-allelic variants of *ATP1A3*). For several of the reported phenotypes additional evidence is required to correlate clinical features with a specific variant. On the other hand, the reporting of atypical features (i.e., childhood onset schizophrenia) may depend on the experience of the clinician who report the case and their familiarity with those features. Moreover, as many of the phenotypes reported are based on retrospective analyses, there might be some inaccuracies regarding precise characterization. The follow-up time of cases reported is also essential, as the expression of certain clinical features is usually age-related. All these elements must be taken into consideration when discussing the genotype-phenotype correlation in *ATP1A3* variants.

Classical *ATP1A3*-related syndromes (e.g., AHC, RDP, CAPOS) manifest significant differences in their prototypical clinical pattern and type of progression.

For example, AHC typically evolve with paroxysmal attacks of weakness. On the other end, RDP evolve usually with no paroxysms and a stationary evolution. Remarkably, among the overlapping syndromes or novel phenotypes, some of them manifest no evident paroxysmal episodes, i.e., CP (p.Pro775Leu), ROA (p.Gly316Ser), COS/ASD (p.Val129Met, p.Asp801Asn, p.Glu815Lys, p.Glu815Lys), SPCA (p.Met154Val, p.Asp350Lys).

*ATP1A3*-related syndromes can be differentiated by severity. Those considered most severe phenotypes are defined by onset in infancy and include AHC and EE. Milder phenotypes have onset in children and adults and include CAPOS, RECA/FIPWE, ROA, and RDP (75, 76).

Genetic heterogeneity and the wide range of phenotypes and disease severity seen with *ATP1A3* variants is not yet understood. To date, almost all disease causing *ATP1A3* pathogenic variants are heterozygous, and when tested usually had loss of function or altered kinetic properties. One exception is the case included in this review, reported by Alloco et al. of a patient compound heterozygous carrying p.Arg19Cys and p.Arg463Cys. This patient presented with severe malformation of the central nervous system (68).

Clinical differences cannot been explained simply by anatomical distribution of ATP1A3, since in animal models this protein is expressed widely in neurons of the CNS and other tissues. However, neuropathologic studies suggest a possible contribution of regional neuronal degeneration and interneuron dysfunction in the mechanism of disease (52). Moreover, MRI studies in some patients with more severe phenotypes showed structural abnormalities (mainly cerebellar atrophy), which might suggest different susceptibility of certain central nervous system areas to functional or structural defects in Na^+^/K^+^ ATPase related with different variants.

Most common missense pathogenic variants are located mainly at highly conserved amino acid residues and seemed to interfere with ATPase activity (77). Pathogenic variants might have detrimental effect on pump activity, ion affinity, ion leakage, or biosynthesis (75). Besides, genetically defined loss-of-function pathogenic variants (frameshifts, premature stops, and deletions) are almost absent from gnomAD for *ATP1A3* (75). On the other hand, a temperature-sensitive gain-of-function mechanism has been postulated to underlie the phenotypic consequences of disease-causing pathogenic variants in *ATP1A3* and its association with environmental triggers. However, the impact of temperature on the functional effects of AHC-and RDP-associated pathogenic variants in ATPase is unknown (8). Sweadner et al. showed that milder phenotypes in *ATP1A3* had a spread distribution, with almost no variants in the ion binding site. In contrast, the variants with severe phenotypes in *ATP1A3* were clustered around the ion binding sites. For the latter, they postulated a gain-of-function with a potential toxic effect by forming larger leaks or outward proton currents because of defective gating (75). In CAPOS the *ATP1A3* p.Glu818Lys variant affects sodium binding to, and release from, a sodium-specific cytoplasmic-facing sites of the Na^+^/K^+^ ATPase. This variant affect the structure of the C-terminal region. It is presumed that these changes affect propagation of membrane potential along the spiral ganglion neurons (41, 78).

Functional analysis of *ATP1A3* pathogenic variants in RDP by haplo-insufficiency determined low protein levels of the corresponding ATPase (13). On the other hand, none of the variants associated with AHC reduced protein levels, whereas both pathogenic variants of AHC and those of RDP reduced ATPase activity (31). These studies suggested that AHC related variants compromise the Na^+^/K^+^ ATPase function due to inhibition of ion binding.

Different phenotype severities have been reported among AHC related pathogenic variants. In general, cases with p.Asp801Asn and p.Gly947Args have a better clinical outcome than p.Glu815Lys carriers. A dominant negative mechanism has been proposed for these heterozygous variants in patients with AHC. According to Li et al. all these pathogenic variants inhibit wild type function by dominant negative interactions in a similar extent, therefore this mechanism is unlikely to explain the AHC severity spectrum (79).

Paradoxically, the severity of human symptoms have not been correlate with whether there was enough residual ATPase activity to support cell survival. Arystarkhova et al. proposed that protein misfolding and endoplasmic reticulum retention were correlated with clinical severity (76).

**Figure 2:**
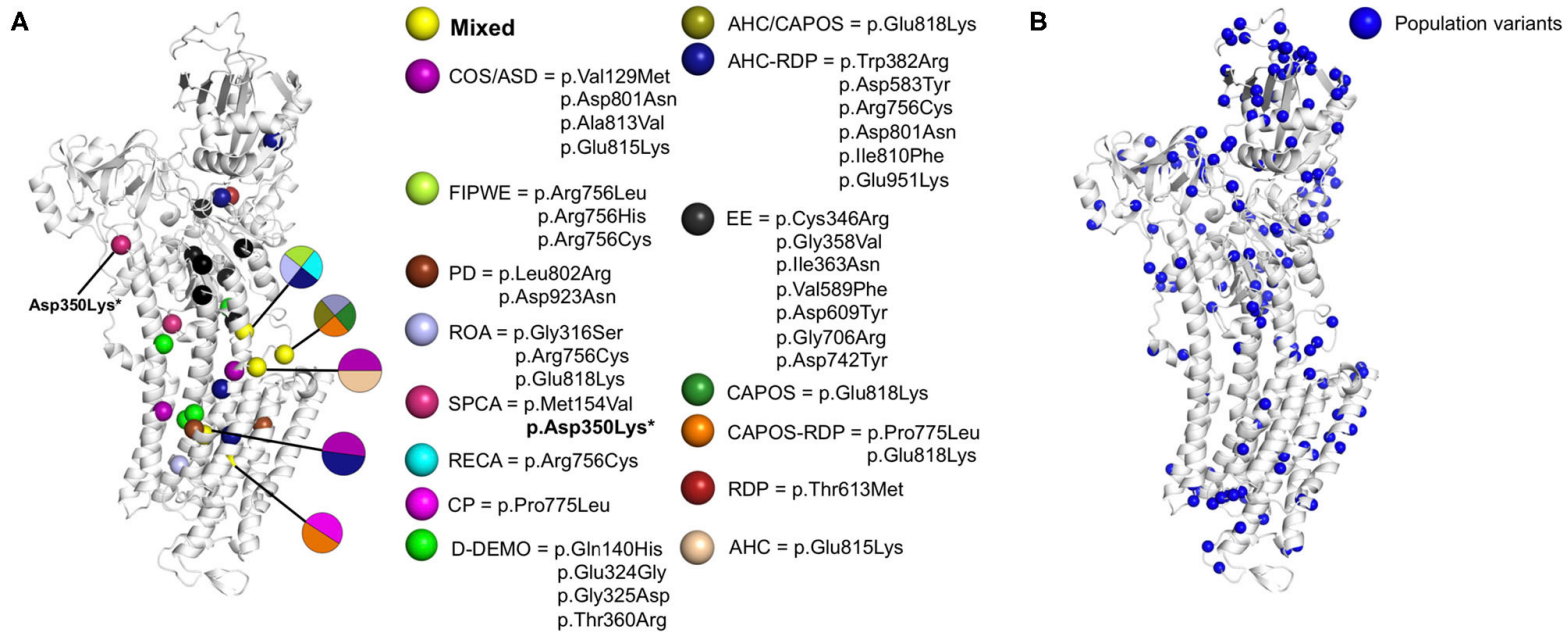
ATP1A3 missense variants mapped on a protein structure model of the α3 subunit of the Na+/K+-ATPase. The homology model was obtained from the SWISS-Model repository (template: 4RET). **(A)** Pathogenic missense variants. Spheres are colored by disorder type. Residues associated with multiple distinct disorders were colored in yellow. **(B)** Population variants in *ATP1A3* were collected from the gnomAD database (81) and visualized as blue spheres on the homology model. *Asp350Lys did not match any protein isoform in Uniprot (82), could not be aligned to the canonical *ATP1A3* sequence and is thus not displayed in the figure. In case of AHC and RDP, we only included the pathogenic variants reported as more frequent according to the references (8, 14), respectively. AHC, alternating hemiplegia of childhood; ASD, autistic spectrum disorder; CAPOS, cerebellar ataxia, areflexia, pes cavus, optic atrophy, and sensorineural hearing loss; COS, childhood-onset schizophrenia; CP, cerebral palsy; D-DEMO dystonia, dysmorphism, encephalopathy, MRI abnormalities, and no hemiplegia; EE, early life epilepsy; FIPWE, fever-induced paroxysmal weakness and encephalopathy; PD, paroxysmal dyskinesias; RECA, relapsing encephalopathy with cerebellar ataxia; RDP, rapid-onset dystonia-parkinsonism; ROA, rapid onset cerebellar ataxia; SPCA, slowly progressive cerebellar ataxia.

We carried out mapping of the variants discussed in the present review. All patient missense variants were visualized on a protein homology model (SWISS-Model repository, template: 4RET) (80) of the α3 subunit of the Na+/K+-ATPase together with control variants in the general population (Figure 2; see methods in Supplementary Material for details). Upon visual inspection, most patient variants were located more near to the center of the protein, whereas population variants tended to be localized more outside the transmembrane helices in the outer cytosolic region. Variants associated with epileptic encephalopathy tended to cluster together in the cytosolic region that is linked to the transmembrane region. These results match those of a similar analysis performed in 2019 comparing variants in three ATP1 paralogs *ATP1A1, ATP1A2*, and *ATP1A3* (75). Notably, all observations could not be statistically quantified likely due to the small number of patient variants and heterogeneity in the variant localization on protein structure.

**Figure 3:**
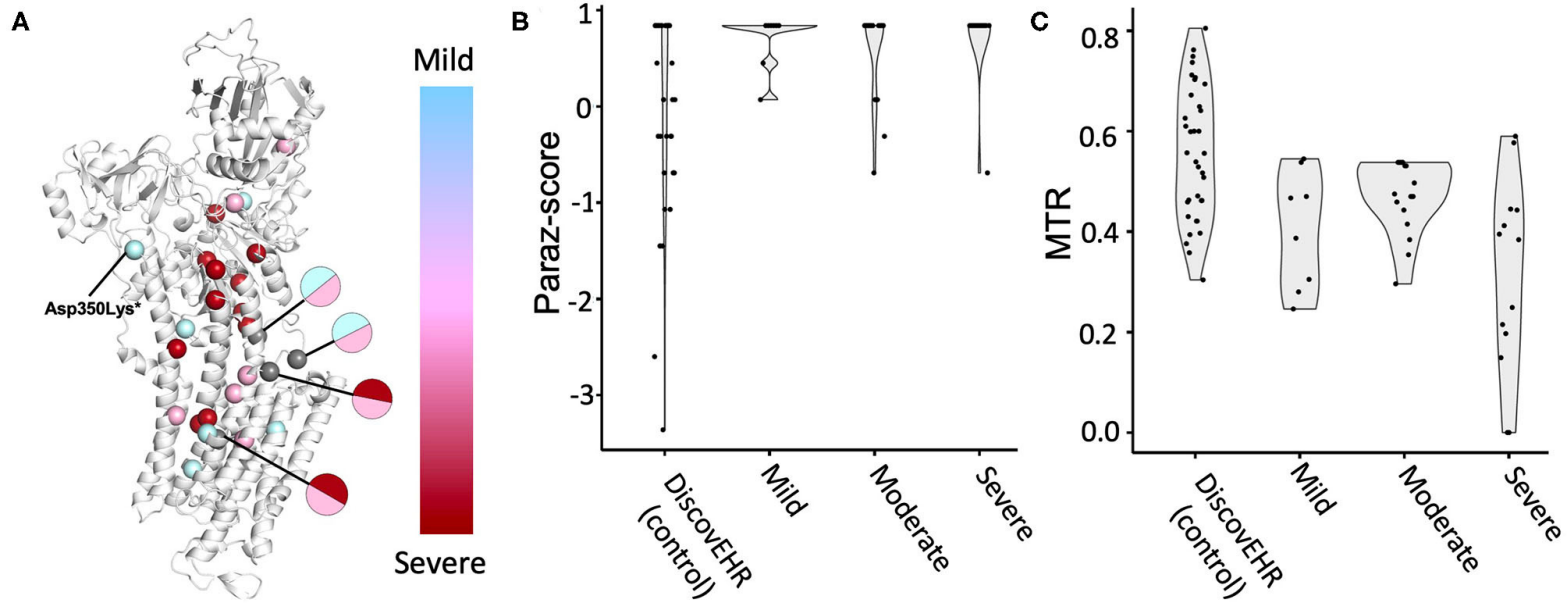
ATP1A3 variant associated disorder severity and variant position analysis. Patients with *ATP1A3* variants were grouped by disorder severity into three groups. **(A)** The corresponding missense variants were visualized on the protein structure and colored according to grouping (mild disorder = cyan; moderate disorder = pink; severe disorder = red). Gray-coloring of spheres indicates residues where variants from multiple severity groups have been reported. Amino acid residue paralog conservation **(B)** and population constrained **(C)** were assessed for variants from each severity group together with a neutral comparison group, missense variants from the DiscovEHR database (see methods in Supplementary Material for details).

We also investigated whether patient variants were located at more evolutionary conserved or population constrained regions compared to a comparison group and explored the localization of the variant severity groups on protein structure spatially (Figure 3A; see methods in Supplementary Material for details). Although more variants associated with more severe disorders tend to be located near the core of the protein, no clear clusters were observed. Amino acids with patient variants were more conserved across paralogous genes compared to the comparison group (*p* = 0.0075). Similarly, patient variants are more constrained to variants from the general population (*p* = 0.0081) (Figures 3B,C). Next, we investigated whether variants associated with mild to severe disorders show differences in evolutionary conserved or population-constrained scores. However, no significant difference was observed as previously described (75).

The available information suggest that different molecular mechanism and complex interactions are involved in the expansive range of disease severity and clinical manifestations.

Most known patients with *ATP1A3*-related disorders fit into discrete classical syndromes with no causal variant overlapping. However, several cases show atypical features or combine features of two or more of these major phenotypes. Moreover, some pathogenic variants have been reported to manifest different phenotypes in non-related cases as well as intrafamilial. In view of the available evidence, we agree with authors that have proposed to consider *ATP1A3*-related disorders as a clinical continuum rather than distinct entities, with an age-dependent pattern of emergence and progression of different signs and symptoms (11). For example, being EE in the most severe extreme and RDP in the milder.

Despite the expanding spectrum of *ATP1A3* phenotypes some features that may guide the clinician in the diagnosis of *ATP1A3*-related disorders include an acute or rapid onset, triggered by fever or other triggers, progression with paroxysmal episodes of dystonia or attacks of weakness, developmental delay, encephalopathy, epilepsy, dysmorphism, pes cavus, hearing loss, optic atrophy, areflexia, pyramidal signs, or a wide range of movement disorders. Remarkably some of these features might differ in cases with atypical presentations. Clinical identification is important to guide molecular investigations and interpretation.

## Author Contributions

PS conducted the review of literature, and wrote the first manuscript. TB and DL performed all the bioinformatic analysis. IM and HF revised and edited the manuscript. All authors revised and edited the final version of the manuscript and read and approved the final version of the manuscript.

## Conflict of Interest

IM and HF Grants/Research Support. IM has received research support from American Parkinson's Disease Association, Parkinson's Foundation, Michael J. Fox Foundation and NIH/NINDS. IF has received research support from Acorda Therapeutics, Alkahest, Amneal, Biogen, Michael J. Fox Foundation, Movement Disorders Society, NIH/NINDS, Parkinson Study Group, Sunovion, but has no owner interest in any pharmaceutical company. HF has received honoraria from, Cleveland Clinic, Boston University, as a speaker in CME events. HF has received honoraria from Bial Neurology, Biopas, Cerevel, CNS Ratings, Denali Therapeutics, Kyowa Hakko Kirin, Pfizer, Partners Healthcare System, Parkinson Study Group, Revance, Sun Pharmaceutical Industries, Sunovion Research and Development Trust as a consultant. Elsevier as the Co-Editor-In-Chief of Parkinsonism and Related Disorders Journal. Royalty: HF has received royalty payments from Demos Publishing and Springer for serving as a book author/editor. Contractual Services: The Cleveland Clinic has a contract with Teva for HF role as a Co-Principal Investigator in Deutetrabenazine for Tardive Dyskinesia global studies. DL receives funds from NIH NINDS, Friends of FACES, German Research Foundation, Federal Ministry of Education and Research (BMBF). The remaining author declares that the research was conducted in the absence of any commercial or financial relationships that could be construed as a potential conflict of interest.
