# Supplementary material for: ATP1A3-Related Disorders: An Ever-Expanding Clinical Spectrum
Source: Front Neurol. 2021 Apr 1;12:637890. doi: 10.3389/fneur.2021.637890 (PMC8047318; doi:10.3389/fneur.2021.637890)
Supplement: Supplementary file 1 [file Data_Sheet_1.docx]

Supplementary Material

# Supplementary Data

**VARIANT MAPPING AND CONSERVATION/CONSTRAINT ANALYSIS**

**A. METHODS**

**A.1. PROTEIN STRUCTURE ANALYSIS**

A molecular solved protein structure for ATP1A3 has not been published or deposited in the protein structure database [1]. We obtained a protein structure model from the SWISS-Model [2] webserver (template = 4ret, chain A, Qmean = -1.83) that covers the residues 18-1013 (97%) of the protein. The patient’ variants listed in this review as well as variants from the general population obtained from the genome aggregation database (gnomAD v2.1.1) [3] were visualized in Pymol (The PyMOL Molecular Graphics System, Schrödinger, LLC. https://pymol.org/)

**A.2. POPULATION CONSTRAINT ANALYSIS.**

To assess the constraint for population variants, the missense tolerance ratio (MTR) [4] was obtained from the MTR-viewer website (http://biosig.unimelb.edu.au; accessed Feb/2021). As a neutral comparison group, we collected variants from the DiscovEHR database (http://www.discovehrshare.com; accessed Feb/2021). DiscovEHR is a variant repository containing more than 50,000 whole exome sequences of patients mainly associated with cardiometabolic, respiratory, and cancer phenotypes. Rare severe pediatric neurodevelopmental disorders were not ascertained in DiscoverEHR. We do not expect pathogenic *ATP1A3* variants in this dataset. The MTR score was generated using gnomAD variants. To avoid a bias due to variants in both datasets, all variants also present in gnomAD were removed from the DiscovEHR control group. Finally, all patient variants were grouped according to their severity into three distinct groups (mild, moderate and severe). This categorization was based on previous reports and on the opinion of the authors. Those phenotypes with early presentation, associated with severe encephalopathy, refractory epilepsy, structural abnormalities of the nervous system, more severe progression, severe neurological compromise between attacks, or early death were considered severe. At the other end, those with an older age at presentation, stationary evolution, less neurological compromise between attacks or less expected functional compromise, were considered mild. For the detailed statistical assessment see the statistical analysis section.

**A.3. PARALOG CONSERVATION AND POPULATION CONSTRAINT ANALYSIS**

To quantify the amino acid conservation across paralogous genes we annotated the Paraz-score. As defined by Lal et al. 2020, genes with >80% sequence similarity are considered to be paralogs [1]. According to this definition, the *ATP1A3* gene family consists out of 6 paralogues genes (*ATP1A3, ATP1A1, ATP1A2, ATP1A4, ATP1A12, ATP4A*). To compare the paralog conservation of the patient variants with a control group, variants from DiscovEHR were annotated (see Population constraint analysis section). Finally, paralog conservation across different levels of severity and the control group were quantified (see statistical analysis section).

**A.4. STATISTICAL ANALYSIS**

A Mann-Whitney-U test was performed to test for differences in the paralog conservation and variant constraint between the different classes of severity and the control variants collected from the DiscovEHR database (see population constraint analysis for details). Visualizations were generated in R (version 4.0.3) with the ggplot2 package (version 3.3.2).

**REFERENCES**

[1] D. Lal, P. May, E. Perez-Palma, K.E. Samocha, J.A. Kosmicki, E.B. Robinson, R.S. Møller, R. Krause, P. Nürnberg, S. Weckhuysen, P. De Jonghe, R. Guerrini, L.M. Niestroj, J. Du, C. Marini, J.S. Ware, M. Kurki, P. Gormley, S. Tang, S. Wu, S. Biskup, A. Poduri, B.A. Neubauer, B.P.C. Koeleman, K.L. Helbig, Y.G. Weber, I. Helbig, A.R. Majithia, A. Palotie, M.J. Daly, Gene family information facilitates variant interpretation and identification of disease-associated genes in neurodevelopmental disorders, Genome Med. 12 (2020) 28. https://doi.org/10.1186/s13073-020-00725-6.

[2] K.J. Karczewski, L.C. Francioli, G. Tiao, B.B. Cummings, J. Alföldi, Q. Wang, R.L. Collins, K.M. Laricchia, A. Ganna, D.P. Birnbaum, L.D. Gauthier, H. Brand, M. Solomonson, N.A. Watts, D. Rhodes, M. Singer-Berk, E.M. England, E.G. Seaby, J.A. Kosmicki, R.K. Walters, K. Tashman, Y. Farjoun, E. Banks, T. Poterba, A. Wang, C. Seed, N. Whiffin, J.X. Chong, K.E. Samocha, E. Pierce-Hoffman, Z. Zappala, A.H. O’Donnell-Luria, E.V. Minikel, B. Weisburd, M. Lek, J.S. Ware, C. Vittal, I.M. Armean, L. Bergelson, K. Cibulskis, K.M. Connolly, M. Covarrubias, S. Donnelly, S. Ferriera, S. Gabriel, J. Gentry, N. Gupta, T. Jeandet, D. Kaplan, C. Llanwarne, R. Munshi, S. Novod, N. Petrillo, D. Roazen, V. Ruano-Rubio, A. Saltzman, M. Schleicher, J. Soto, K. Tibbetts, C. Tolonen, G. Wade, M.E. Talkowski, B.M. Neale, M.J. Daly, D.G. MacArthur, The mutational constraint spectrum quantified from variation in 141,456 humans, Nature. 581 (2020) 434–443. https://doi.org/10.1038/s41586-020-2308-7.

[3] The UniProt Consortium, UniProt: a worldwide hub of protein knowledge, Nucleic Acids Res. 47 (2019) D506–D515. https://doi.org/10.1093/nar/gky1049.

[4] H.M. Berman, J. Westbrook, Z. Feng, G. Gilliland, T.N. Bhat, H. Weissig, I.N. Shindyalov, P.E. Bourne, The Protein Data Bank., Nucleic Acids Res. 28 (2000) 235–42. https://doi.org/10.1093/nar/28.1.235.
